# Region Specific Effects of Aging and the Nurr1-Null Heterozygous Genotype on Dopamine Neurotransmission

**DOI:** 10.4172/2469-9780.1000114

**Published:** 2017-04-05

**Authors:** Evangel Kummari, Shirley Guo-Ross, Jeffrey B Eells

**Affiliations:** Department of Basic Sciences, College of Veterinary Medicine, Mississippi State University, Mississippi State, MS 39762, USA

**Keywords:** NR4A2, Parkinson’s disease, Dopamine

## Abstract

The transcription factor Nurr1 is essential for dopamine neuron differentiation and is important in maintaining dopamine synthesis and neurotransmission in the adult. Reduced Nurr1 function, due to the Nurr1-null heterozygous genotype (+/−), impacts dopamine neuron function in a region specific manner resulting in a decrease in dopamine synthesis in the dorsal and ventral striatum and a decrease in tissue dopamine levels in the ventral striatum. Additionally, maintenance of tissue dopamine levels in the dorsal striatum and survival of nigrostriatal dopamine neurons with aging (>15 months) or after various toxicant treatments are impaired. To further investigate the effects of aging and the Nurr1-null heterozygous genotype, we measured regional tissue dopamine levels, dopamine neuron numbers, body weight, open field activity and rota-rod performance in young (3–5 months) and aged (15–17 months) wild-type +/+ and +/− mice. Behavioral tests revealed no significant differences in rota-rod performance or basal open field activity as a result of aging or genotype. The +/− mice did show a significant increase in open field activity after 3 min of restraint stress. No differences in tissue dopamine levels were found in the dorsal striatum. However, there were significant reductions in tissue dopamine levels in the ventral striatum, which was separated into the nucleus accumbens core and shell, in the aged +/− mice. These data indicate that the mesoaccumbens system is more susceptible to the combination of aging and the +/− genotype than the nigrostriatal system. Additionally, the effects of aging and the +/− genotype may be dependent on genetic background or housing conditions. As Nurr1 mutations have been implicated in a number of diseases associated with dopamine neurotransmission, further data is needed to understand why and how Nurr1 can have differential functions across different dopamine neuron populations in aging.

## Introduction

Dopamine neurotransmission has been implicated in a number of pathological conditions including Parkinson’s disease, schizophrenia, attention deficit hyperactivity disorder and addiction [1–4]. Nurr1 (NR4A2) is a nuclear receptor/transcription factor that is essential for proper development of mesencephalic dopamine neurons as homozygous disruption of Nurr1 stops differentiation of these neurons [5–7]. Nurr1 is the upstream regulator of genes involved in the synthesis, packaging, transport and reuptake of Dopamine [8,9] Overexpression of Nurr1 and Pitx3 in mouse induced pluripotent stem cells can program them into functional dopaminergic like neurons [10].

Several intrinsic mechanisms have been identified in the mesencephalic DA neurons that are linked to Nurr1 mediated cell survival [11–14]. *In vitro* and *in vivo* studies demonstrate that Nurr1 gene delivery/therapy and Nurr1 activation/activating compounds enhance DA as well as protect mesencehpalic dopaminergic neurons from cell injury induced by toxins or neuroinflammation and nigrostraiatal associated motor behaviors of dopamine neurotransmission [15–18]. Nurr1 is also neuroprotective in nature as it inhibits the expression of Pro-inflammatory neurotoxic mediators in microglia and astrocytes by recruiting CoREST corepressor complex thereby preventing the loss of dopaminergic neurons [19,20].

Mutation analysis has implicated a role for Nurr1 in some of these pathological conditions. Mutations in Nurr1 have been linked to Parkinson’s disease [21–24] and Nurr1 is reduced in patients with Parkinson’s disease and correlates with the loss of tyrosine hydroxylase immunoreactivity [25–27]. Two different missense mutations in exon 3 of Nurr1 were identified in 3 patients with either schizophrenia or bipolar disorder [28].

Nurr1 has been implicated as a transcription factor that regulates the expression of several dopamine neurotransmission genes including tyrosine hydroxylase, dopamine transporter, vesicular monoamine transporter and GTP cyclohydrolase [9,26,29–33]. With the potential to alter multiple parameters regulating dopamine neurotransmission, the effects of Nurr1 on dopamine neurotransmission is complex. The role of Nurr1 in the regulation of dopamine neurotransmission in adult animals is mostly based on experiments in the Nurr1-null heterozygous mice (+/−). The Nurr1 +/− genotype has been shown to have a subtle but significant effect on dopamine neuron function in the nigrostriatal dopamine system [31,34]. Although the Nurr1 +/− mice have normal numbers of nigrostriatal dopamine neurons and dopamine levels in the striatum, these mice have reduced tyrosine hydroxylase activity and an apparently reduced capacity to maintain dopamine levels [31]. Additionally, reduced Nurr1 function in these mice increases the susceptibility of nigrostriatal dopamine neurons to the neurotoxins MPTP, amphetamine and rotenone and the irreversible proteasome inhibitor lactacystin [35–37]. Similarly, when dopamine neurons from +/+ and +/− newborn pups were grown in culture, survival and neurite growth in dopamine neurons from +/− mice was significantly reduced [38]. Although the nigrostriatal dopamine system is impacted by the Nurr1 +/− genotype, the mesoaccumbens dopamine system (i.e., dopamine neurons in the substantia nigra pars Compacta and ventral tegmental area) that innervate the ventral striatum consisting of the nucleus accumbens core and shell (NAC and NAS, respectively) appears to be more susceptible to the effects of the +/− genotype. Significant reductions in tissue dopamine levels in the nucleus accumbens and GTP cyclohydrolase mRNA expression in the ventral tegmental area have been reported [31,39,40] No difference in these parameters were observed in nigrostriatal system [31,39,40] Additionally, a significant elevation in synaptic dopamine levels, as measured by microdialysis was found in the shell of the nucleus accumbens of Nurr1 +/− mice that was not observed in the striatum [41]. Previous studies have found that aging is an important parameter that affects both dopamine neurotransmission and Nurr1 levels. Aging produces various changes in the function of the nigrostriatal dopamine neurotransmission; however, the mechanisms of these changes are unclear. A number of changes in dopamine function have been reported in the striatum. One of the most consistent is the decrease in D2 receptor expression in the striatum [42]. In the Nurr1 +/− mice, reductions in striatal dopamine levels, reduced numbers of dopamine neurons and a decrease in rota-rod performance in the aged (>9 months) +/− mice have been reported [34,43]. Based on these data, the Nurr1 +/− mice may represent a potentially useful model of Parkinson’s disease because it combines a genetic mutation that increases the susceptibility of the nigrostriatal dopamine neurons to an environmental stressor such as aging Which mimics parameters thought to contribute to Parkinson’s disease. These data suggest that aging can influence Nurr1 expression and also produces various changes in the function of the nigrostriatal dopamine neurotransmission. It is unclear, however, how aging could also affect other dopamine systems particularly the mesoaccumbens system which could provide insight into the differences in regulation between these neuron populations. These experiments were initiated to further examine the effects of aging in the Nurr1 +/− mice on both the nigrostriatal and mesoaccumbens dopamine systems. The effect of the combination of aging and the Nurr1 +/− genotype on extracellular dopamine levels and dopamine release in the striatum has not been reported. Furthermore, these studies were investigated to also determine if aging has similar or distinct effects on the mesoaccumbens dopamine neurons associated with aging. The data indicate that aging and the +/− genotype can produce subtle effects on nigrostriatal dopamine neurotransmission. However, the regulation of tissue dopamine levels in the ventral striatum is the most susceptible to the combination of aging and the +/− genotype.

## Materials and Methods

### Chemicals and reagents

Quinpirole, standards for high performance liquid chromatography (HPLC) analysis including dopamine, 3,4-dihydroxyphenylacetic acid (DOPAC) and homovanillic acid (HVA) and HPLC reagents were purchased from Sigma- Aldrich (St. Louis, MO). CNS perfusion fluid was purchased from CMA Microdialysis (North Chelmsford, MA). Reagents for the HPLC mobile phase were purchased from Sigma-Aldrich (St. Louis, MO).

### Animals and guidelines

The Nurr1-null heterozygous mice used for this study were obtained from a colony bred at Mississippi State University originally produced in the laboratory of Dr. Vera Nikodem at the National Institute for Diabetes and Digestive and Kidney Diseases [6]. Mice were genotyped as previously described to distinguish +/− and +/+ mice [6]. Litters were chosen at random for either young or aged mice. At 19–21 days of age, mice were weaned and housed in groups of 3–5/cage. Mice were housed in cages with steel grid lids and all cages were located in the same room. All procedures were performed in accordance with the National Institutes of Health Guide for the Care and Use of Laboratory Animals, and study protocols were approved by the Institutional Animal Care and Use Committee at Mississippi State University. All animals used in this project were housed in the AAALAC accredited facilities of the College of Veterinary Medicine, Mississippi State University. The individual room temperatures were maintained between 18–22°C with food and water *ad libitum*. Care of the mice was overseen by a laboratory animal veterinarian. Male mice were used for behavior analysis, immunohistochemistry, and neurochemistry measurements.

### Behavior

To assess motor coordination, mice were tested using a 4 station rota-rod treadmill for mice (Med Associates, St. Albans, VT). The rota-rod was set to increase rotation speed from 3–30 rpm. Each mouse underwent 2 rounds of training. The mice were placed on the rod for 2 min. If the mouse fell off during this time it was placed back on the treadmill so that all mice received the same amount of training time. Each mouse had a rest time 4 min in between each training round. After the training, 3 test trials were done on each mouse. The time spent and the speed reached by each mouse was recorded and compared between +/+ and +/− young and aged mice. At the end of rota-rod testing, mice were weighed. To assess spontaneous and stress induced locomotor activity, mice were placed in an open field chamber consisted of a 25 cm × 25 cm plexiglass enclosure with a video camera mounted above and attached to a computer containing the LimeLight software to measure total distance traveled. The basal activity of the mice is monitored initially for 45 min by a video camera. After this 45 min activity period, mice were placed in a Broome rodent restrainer for 3 min to produce restraint stress, then the mice were put back into the open field chamber and the activity was monitored for another 45 min. The total distance traveled during the basal condition and the stress condition was compared across age and genotype. The number of mice used for behavior analysis included 22 +/+ young, 37 +/− young, 7 +/+ aged, and 8 +/− aged.

### Tissue dissection

One week after the open field test, the mice were euthanized with CO_2_ asphyxiation, the brains removed, cut with a coronal section at approximately 1 mm caudal to Bregma into forebrain and midbrain pieces. The forebrain was further split in half with a sagittal cut. The midbrain piece and the right forebrain piece were immersion fixed in 4% paraformaldehyde for 24 h then placed in 30% sucrose for 2 days. The left forebrain piece was frozen on dry ice and stored at −80°C.

### Catecholamine isolation

**Figure 1:**
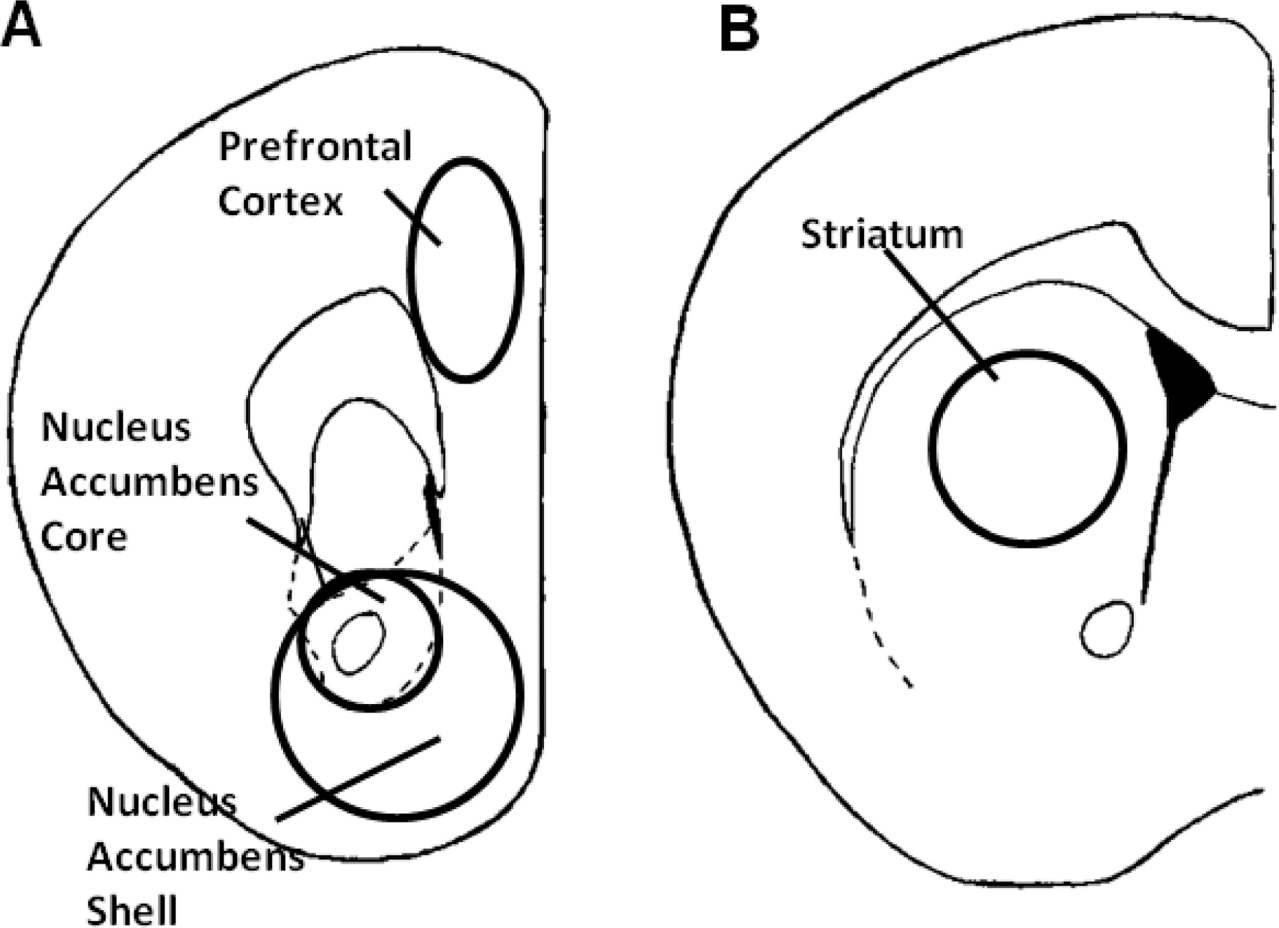
Approximations of the regions and size of tissue dissected in thick frozen section and used for dopamine neurochemistry: Sections are approximately 1.70 mm (A) and 1.00 mm (B) rostral to Bregma. Sections were modified from Paxinos and Franklin.

The left piece of frozen forebrain tissue was mounted in a custom made tissue slicer with OCT compound (Sakura Finetek, Torrance, CA) and 600–800 µm frozen sections were cut and mounted on glass slides. Micropunches of the dorsal striatum were isolated using a blunt 20 gauge needle. Micropunches of the nucleus accumbens core was taken with a 22 gauge blunt needle then the remaining nucleus accumbens shell was dissected using an 18 gauge blunt needle. A micropunch of the prefrontal cortex was also taken with a blunt 20 gauge needle. Approximate locations of these dissected regions are shown on tyrosine hydroxylase immunohistochemistry sections in Figure 1. Micropunches were used for determination of dopamine and metabolite levels using high performance liquid chromatography (HPLC) and electrochemical detection. Micropunches were sonicated in 0.1 M perchloric acid and 100 Μm EDTA at 4°C then cleared by two successive centrifugations at 10,000 g. The cleared supernatant was injected into a HPLC system consisting of a Waters 2695 Separation module. The remaining pellet was solubilized in 1M HCl and total protein was determined using BCA according to manufactures instructions. The number of male mice used for tissue dopamine levels consisted of 7 +/+ young, 10 +/− young, 7 +/+ aged, and 8 +/− aged.

### Catecholamine measurements

Tissue extractions (10–20 µL) or microdialysis fractions (18 µL) were injected into a HPLC system consisting of a Waters 2695 Separation module, a SupleCosil LC-18-DB column with the Waters 2465 electrochemical detector set at 20 nA and an Ec=+0.67 V using a mobile phase of 100 mM phosphate, 17.5% methanol, 25 µM EDTA, 1 mM octyl sodium sulfate at pH 3.65. The quantity of each compound was determined based on the response of a known amount of standards of dopamine, DOPAC and HVA (Sigma Aldrich, St. Louis, MO) and are reported as pg in the dialysate.

### Immunohistochemistry and stereology

Fixed midbrain and forebrain tissue was serial sectioned into 30 nm sections. Every 6th section was used for immunhistochemistry. Free-floating sections were washed 3 times in phosphate buffered saline (PBS) with 1% bovine serum albumin (BSA), and then incubated for 30 min in 1% H_2_O_2_ in PBS. Sections were washed three times in PBS then incubated in blocking serum (PBS with 1% Triton-X 100, 4% normal goat serum, 1% BSA) for 30 min. Sections were then incubated in a rabbit polyclonal anti-tyrosine hydroxylase antibody (Millipore, Billerica, MA) diluted 1:5000 at room temperature for 2 h. Sections were rinsed 10 times with PBS containing 1% BSA and 0.2% Triton X-100 then incubated in biotinylated anti-rabbit IgG for 2 hours, rinsed 3 times. Sections were then incubated for 2 h in equilibrated ABC reagent (Vector Laboratories, Burlingame, CA) diluted in PBS-0.02% Triton X-100 and 1.0% bovine serum albumin. Sections were rinsed 2 times in PBS then incubated in a 0.5× diaminobenzidine solution (Sigma-Aldrich, St. Louis, MO) with 0.003% H_2_O_2_ for 5 min. Sections were rinsed in PBS and counterstained with nuclear fast red (Sigma-Aldrich, St. Louis, MO). Sections were mounted onto silanized slides, dehydrated in graded ethanol series followed by xylene, then cover slipped with Permount. Immunoreactivity was evaluated and stereology was performed using an Olympus BX51 microscope with a CCD camera and a motorized Z-stage which is all connected to a computer with Stereo Investigator Stereology Software from MicroBrightField Inc. (Williston, VT). Unbiased stereology on tyrosine hydroxylase immunoreactive profiles in the substantia nigra pars compacta and ventral tegmental area was performed using Stereo Investigator Stereology Software from MicroBrightField Inc. The optical fractionation method was used for estimating tyrosine hydroxylase immunoreactive profiles. The substantia nigra pars compacta and the ventral tegmental area were outlined based on tyrosine hydroxylase labeling. A 60× oil immersion objective was used to count profiles and measure section thickness at each sample site. Stereologic parameters used were a grid size of 150 µm × 150 µm with a random orientation and an optical dissector height of 16 µm. Estimates of immunoreactive profiles were made in young and aged +/+ and +/− mice (n=3/group) in both the substantia nigra pars compacta and ventral tegmental area. The number of mice used for stereological counts consisted of 3 +/+ young, 3 +/− young, 3 +/+ aged, and 3 +/− aged.

## Statistical Analysis

Behavioral data, tissue catecholamine levels and stereological estimates were analyzed using ANOVA with Fisher’s PLSD post-hoc comparison.

## Results

### Rota-rod performance and open field activity in young and aged, +/+ and +/− mice

**Figure 2:**
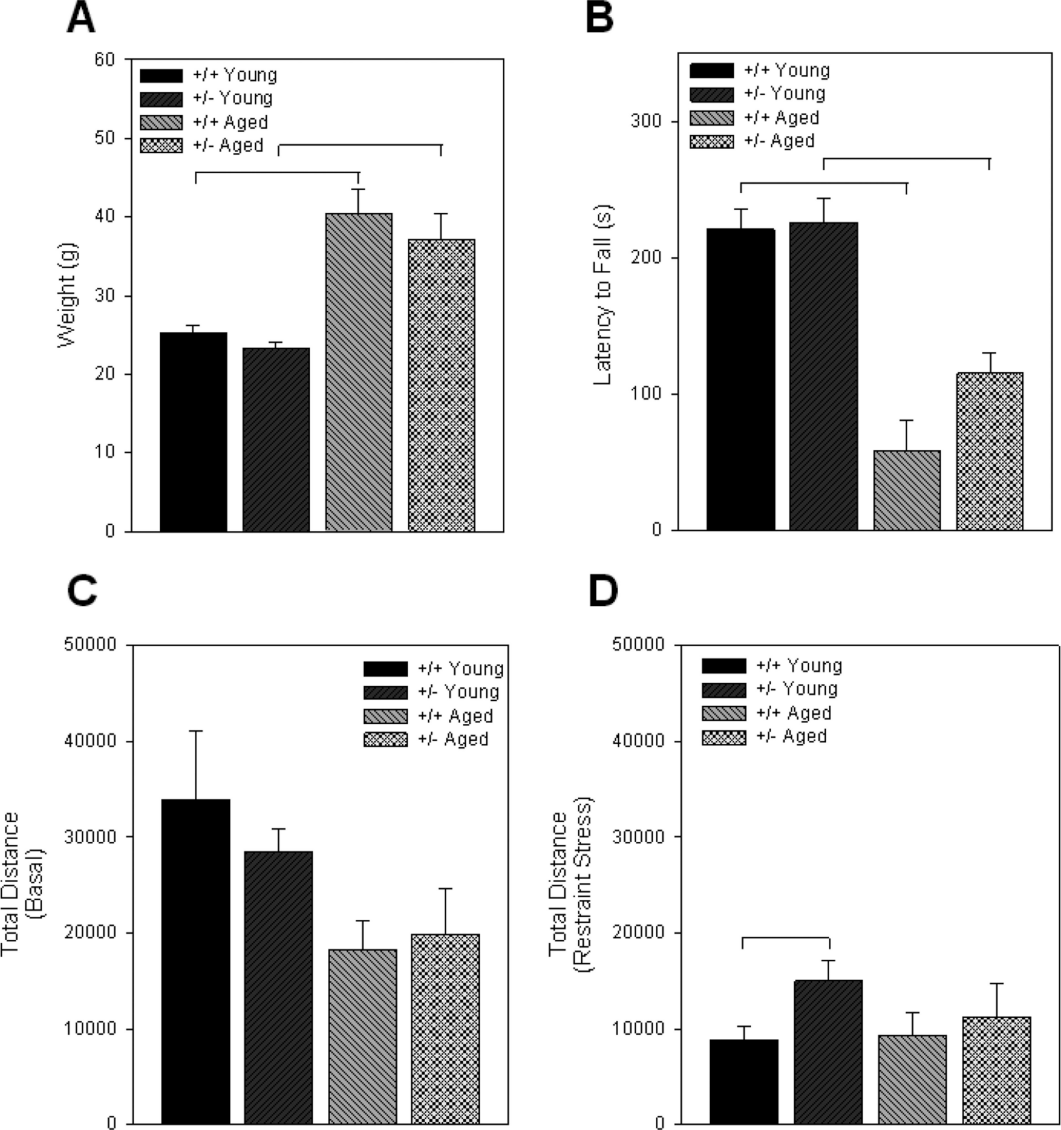
Behavior analysis due to aging: Body weight (A), rota-rod performance (B) and total distance traveled in an open field activity field under basal conditions (C) and after 3 min of restraint stress (D) were measured in young and aged wild-type (+/+) and Nurr1-null heterozygous (+/−) mice. No genotype differences in body weight were found, although aging significantly increased with body weight. Rota-rod performance was also significantly impaired with aging. The aged +/+ mice showed a trend toward impaired rota-rod performance, but was not significantly different compared to the aged +/− mice (C). No significant differences were found in basal open field activity, however, +/− mice were significantly more active after 3 min of restraint stress. Bars represent mean ± S.E.M. Brackets represent significant difference between treatments based on ANOVA with Fisher’s PLSD post-hoc comparison with p<0.05.

Behavioral analysis was carried out on the young and aged, +/+ and +/− mice. The behavioral test consisted of rota-rod performance, open field activity in a novel environment and after 3 min of restraint stress. The weight of each mouse was also recorded. Aging significantly increased weight. Although there was no significant difference across genotype, the +/− mice had slightly lower body weights (Figure 2A). There was a positive correlation between age and weight and a negative correlation between rota-rod performance and weight. In the rota-rod test, aging significantly reduced rota-rod performance in both +/+ and +/− mice (Figure 2B). No significant differences were observed on rota-rod performance due to genotype. There was a trend of reduced performance in the aged +/+ mice but much of this was the result of two animals with very low performance (3.7 and 5.7 s). In open field activity, there were no significant differences in total distance traveled in the basal condition (Figure 2C). The young +/− mice, however, were significantly more active after the restraint stress than the young +/+ mice (Figure 2D). In the aged mice, there was no genotype difference in stress induced activity although there were fewer aged mice tested than young mice.

### Tissue dopamine levels in the ventral striatum are attenuated by aging in the +/− mice

**Figure 3:**
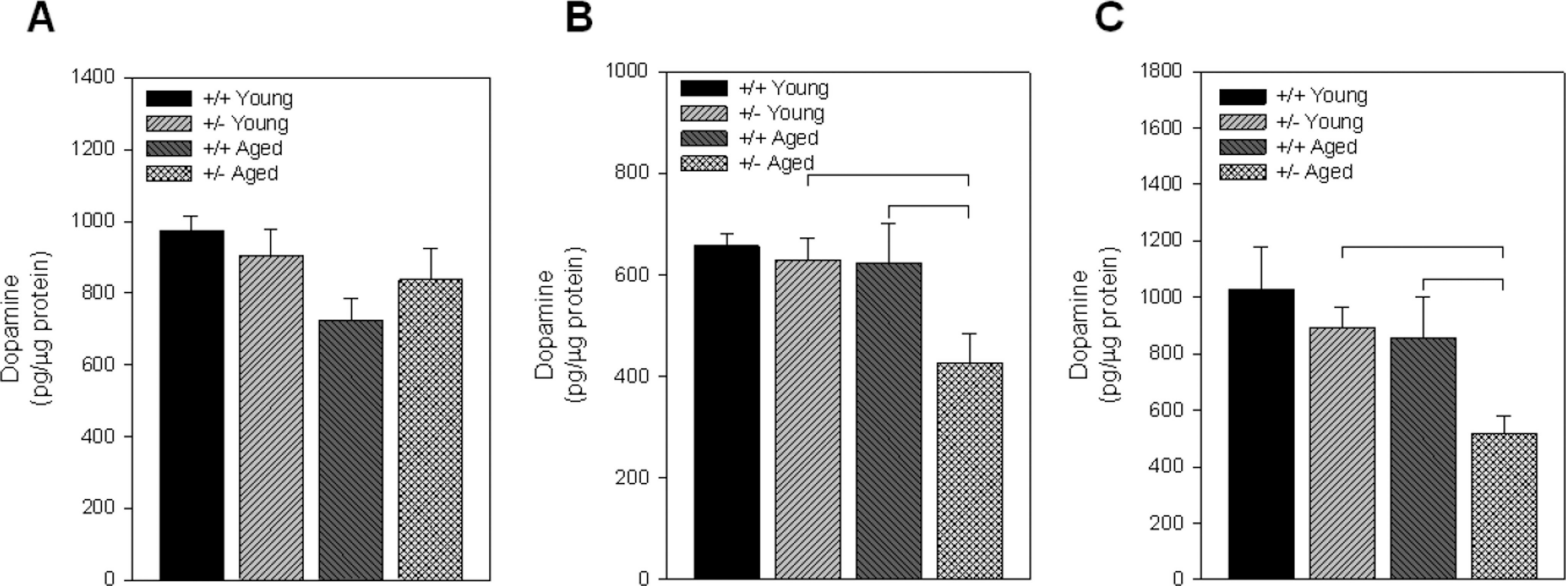
Tissue dopamine levels: Dopamine levels were measured in tissue punches from the dorsal striatum (A), nucleus accumbens shell (B), and n ucleus accumbens core (C) across young and aged wildtype (+/+) and Nurr1-null heterozygous (+/−) mice. There was a significant reduction in dopamine in the nucleus accumbens shell and core in the aged +/− mice. Bar graphs represent mean ± S.E.M. Brackets represent significant difference between treatments based on ANOVA with Fisher’s PLSD post-hoc comparison with p<0.05.

Tissue dopamine and metabolite levels were measured in micropunches from the dorsal striatum, the ventral striatum separated into the NAC and NAS, and the prefrontal cortex across young and aged +/+ and +/− mice. Within the dorsal striatum, there were no significant differences in tissue dopamine levels observed due to the +/− genotype or aging (F3,28=2.692, p=0.0660) (Figure 3A). In fact, there was a trend toward a reduction in tissue dopamine levels from the dorsal striatum in the aged +/+ mice as compared to young +/+ mice and aged +/− mice (Figure 3A). There were no significant differences in DOPAC or HVA levels or dopamine turnover in the dorsal striatum (Table 1). The ventral striatum, consisting of the NAS and the NAC, showed a different pattern of changes in dopamine neurochemistry as compared to the dorsal striatum. Specifically, there was a significant reduction in tissue dopamine levels in the aged +/− mice as compared to both the young +/− mice and the aged +/+ mice similarly in both the NAS (F3,28=2.949, p=0.049) and NAC (F3,28=4.05, p=0.0165) (Figure 3B and 3C). Within the NAC, there was also a significant reduction in DOPAC levels in the aged +/− mice (F3, 28=4.092, p=0.0162) (Table 1). No differences in HVA levels were found across groups in the either the NAC or NAS. There were significant elevations in dopamine turnover (dopamine/HVA) in the aged +/− mice in the NAS and NAC. No differences in dopamine neurochemistry were observed in the prefrontal cortex across age or genotype.

### Dopamine neuron immunohistochemistry found no effects with aging and the +/− genotype on dopamine neuron survival

**Figure 4:**
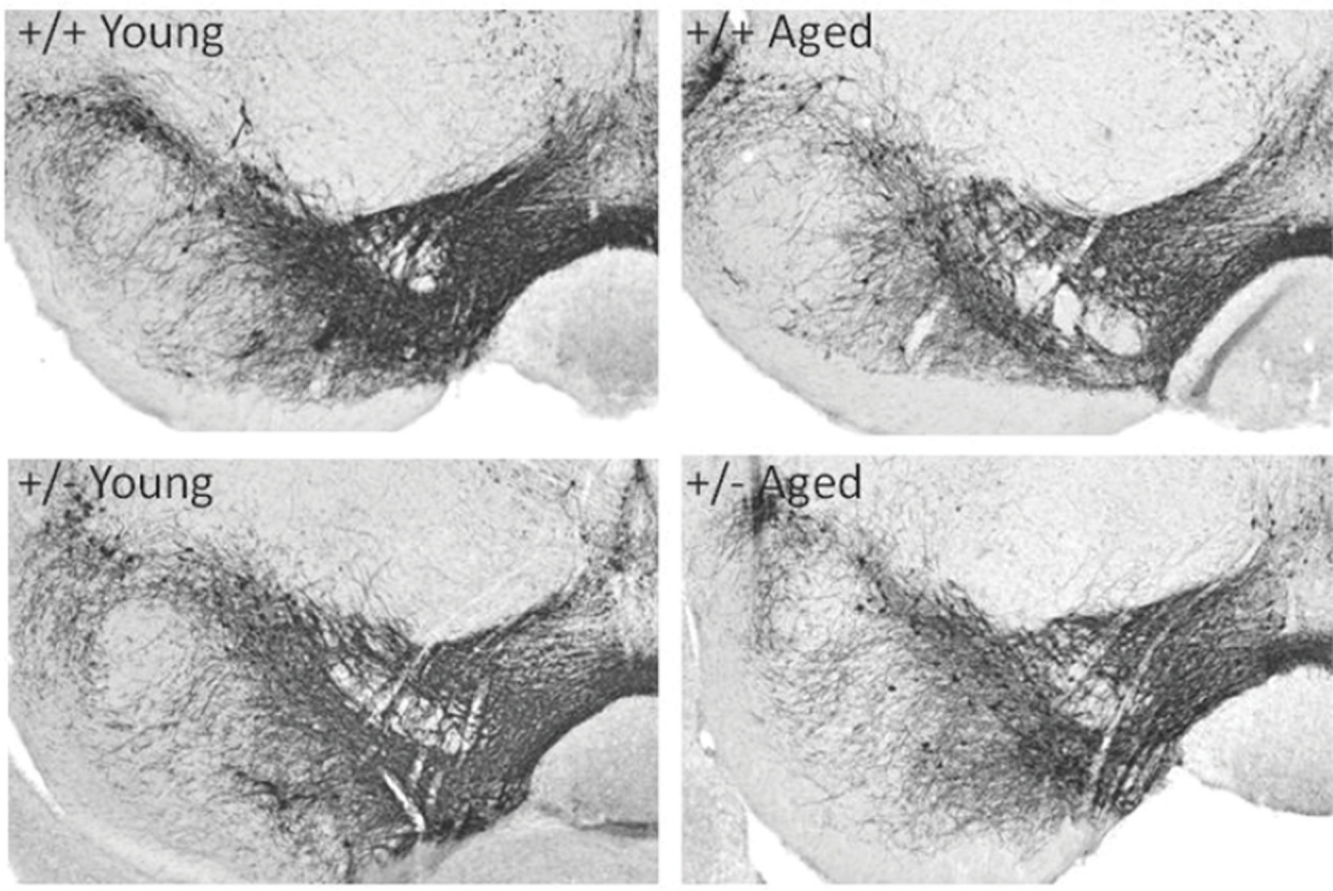
Tyrosine hydroxylase immunohistochemistry: TH immunohistochemistry was examined in the ventral midbrain of young and aged wild-type (+/+) and Nurr1-null heterozyous (+/−) mice. No noticeable differences in distribution of tyrosine hydroxylase immunoreactive neurons were observed across these groups.

Survival and innervations of dopamine neurons was determined across aging and the +/− genotype using tyrosine hydroxylase immunohistochemistry. To determine if aging and the +/− genotype affected survival of dopamine neurons, unbiased stereology was used to estimate dopamine neuron population in the substantia nigra pars compacta and the ventral tegmental area. No noticeable differences in the intensity or distribution of dopamine neurons in either the substantia nigra pars compacts or ventral tegmental area were observed across groups in either of these areas (Figure 4). Unbiased stereology found no differences in the estimated number of tyrosine hydroxylase immunoreactive neurons in either the substantia nigra or ventral tegmental area across age or genotype (Table 2). Target areas of dopamine neuron innervations were also investigated using tyrosine hydroxylase immunohistochemistry. No obvious differences were observed in any of these target areas, dorsal or ventral striatum (data not shown).

## Discussion

Currently, animal models that reproduce all of the neuropathology and neurodegeneration found in Parkinson’s disease are lacking. The most widely used models consist of the use of various toxins to kill or damage dopamine neurons. Because of the important contribution of aging in the etiology of Parkinson’s disease as well as links to genetics, a more ideal model would consist of a genetic mutation that, when combined with aging, produces progressive deficits in dopamine levels and neurodegeneration of nigrostriatal dopamine neurons. Previous reports using aged Nurr1 +/− mice have described significant effects in the nigrostriatal dopamine system. Aging (as early as 9–12 months) in the Nurr1 +/− mice resulted in a significant reduction in dopamine levels in the striatum, impaired performance on the rota-rod, and reduced numbers of dopamine neurons in the substantia nigra pars compacta in the aged +/− mice [34,43]. Because of the potential importance of this model, we began further investigations into how aging alters dopamine transmission in Nurr1 +/− mice but also included other mesencephalic dopaminergic systems. In contrast to previous reports, the current data found no difference in numbers of dopamine neurons or tissue dopamine levels in the dorsal striatum of aged +/− mice. However, significant reductions in tissue dopamine levels in the ventral striatum, including the NAS and NAC, resulted from the combination of aging and the +/− genotype. Potential reasons for these differences could be explained by either the background strain of mice or construct used. Three independently derived strains of Nurr1 knockout mice were produced by the laboratories of Dr. Conneely [8], Dr. Perlmann [5] and Dr. Nikodem [6] and used between the various studies. Comparisons between parameters used to create these different lines have been reviewed previously [44]. Backman et al. using the Perlmann derived mice, reported no differences in tissue dopamine levels using micropunches from the striatum or number of dopamine neurons in the substantia nigra pars compacta in Nurr1 +/− mice 12–15 months of age [45]. In fact, these authors found higher, but not significantly higher, dopamine and DOPAC levels in the +/− mice. These results more closely resemble the data in this current report using the Nikodem line of Nurr1 +/− mice. Jiang et al. and Zhang et al. both found significant reductions in dopamine neuron numbers in the substantia nigra and reduced tissue dopamine in the striatum at 9–12 months [35] and 15–19 months [46] of age in the Nurr1 +/− mice using the Connelly line of Nurr1 +/− mice. Interestingly, [36] reported no differences in tissue dopamine levels in the striatum or the number of nigrostriatal dopamine neurons in aged (13–14 month) Nurr1 +/− mice, also using the Connelly line. More recently, when the construct used here was breed into C57-BL6 mice, there was a significant decrease in body weight and increase in novel open field activity in the Nurr1 +/− mice [47]. This suggests that the background strain could have an impact on the penetrance or expressivity of the Nurr1 +/− mutation to impact survival of dopamine neurons with aging. Although genes have been identified that can cause Parkinson’s disease, such as SNCA, LRRK2, parkin, PINK1, and DJ-1 [48]. most cases have a relatively small genetic component with low concordance rates among monozygotic twins [49–51]. Therefore, understanding the genetic context necessary for the Nurr1 +/− genotype to produce a loss of dopamine neurons and tissue dopamine in the striatum could be very informative. Differences in housing condition or other aspects of the environment may also impact the role of Nurr1 in regulating dopamine neuron function and survival. Nurr1, as an immediate early gene, is sensitive to stress, can be induced by various drugs, and could be sensitive to various housing conditions [41,42,52,53]. Previous data demonstrated that isolation had a significant effect on tissue dopamine levels in the dorsal striatum and that isolation could have a differential effect, depending on the +/− genotype, on dopamine neurotransmission in the NAS [41,42]. In the current study, all mice were raised in groups of 3–5, none were isolated. Differences across laboratories in how the mice were reared, such as when they are weaned or types of caging could also have an effect on the results. Any factor that produces different levels of stressors has the potential to impact Nurr1 expression and dopamine neuron function. Elevated stress induced open-field activity has been consistently reported in the Nurr1 +/− mice. This result was first reported in by Eells et al. and replicated by others and appears to be the most robust behavioral finding in these mice regardless of the derived strain [40,45,46,54,55]. To produce a stress response, we restrained the mice for 3 min prior to placing them back in the open-field. The young +/− mice showed a significant increase in open-field activity after stress. We had previously assigned the stress induced increase in activity to differences in mesoaccumbens dopamine neurotransmission. However, based on the present study, this difference is apparent in the absence of any detected difference in dopamine levels in the nucleus accumbens of the +/− mice. Although alterations in the mesoaccumbens neurotransmission could account for differences in open-field activity in the +/−, the precise mechanism that mediates the difference in the stress induced open-field activity in the +/− mice is unclear. Previous data found that tyrosine hydroxylase activity is reduced in the striatum of the +/− mice and that this difference is enhanced when feedback on the dopamine neuron is block via inhibiting dopamine release [32] This suggests a potential difference in feedback on the dopamine autoreceptor to more closely maintain dopamine synthesis. Although the Nurr1 +/− genotype has been found to have significant effects on the nigrostriatal dopamine system, effects on the mesoaccumbens dopamine system appear to be more prominent although less well characterized. Significant reductions in tissue dopamine levels were reported in the +/− mice in the nucleus accumbens without significant effects in the striatum [40,41]. In the current data, no significant differences in dopamine levels in the ventral striatum of young +/− mice were observed, either in the NAS or NAC. Differences in dissection technique (dorsal and ventral striatum isolation from fresh tissue versus micropunches in frozen sections) and electrochemical detection methods (extraction versus direct measurement) could account for some differences. Aging, however, may be an important variable in producing the deficit in dopamine in the ventral striatum. Additionally, breeding may impact the effect of the +/− mutation as mentioned above. Further studies with direct comparisons at different ages will be important to differentiate effects here. Additionally, the effect aging has on Nurr1 levels in dopamine neurons in the ventral tegmental area have not been investigated. The striatum consists of medium spiny neurons that primarily receive synaptic input from the pyramidal neurons in the cerebral cortex along with dopamine innervations from the mesencephalon. Dopamine innervation to the dorsal striatum consists of dopamine neurons in the substantia nigra pars compacta. The ventral striatum, however, receives dopamine innervations primarily from the ventral tegmental area, mostly the NAS. Dopamine neurons in the medial substantia nigra pars compacta and lateral ventral tegmental area innevate the NAC [56]. Studies have found differences in electrophysiology and gene expression between nigrostriatal dopamine neurons in the substantia nigra pars compacta and mesoaccumbens dopamine neurons in the ventral tegmental area [57,58]. Differences in autoreceptor function and/or dopamine uptake between these areas could underlie the observed effects of aging and the +/− genotype between the dorsal and ventral striatum. Currently, how Nurr1 can differentially affect these separate dopamine systems has not been elucidated. It is unclear whether these differences are due to differences in the neurons innervating these areas or whether there are local effects across the dorsal and ventral striatum that result in the differences in tissue dopamine levels observed.

## Conclusions

The Nurr1 +/− genotype appears to be an important regulator of tissue dopamine as it relates to the mesoaccumbens dopamine system and that aging is an important variable in how Nurr1 regulates dopamine levels. As for the nigrostriatal dopamine system, there are, apparently, other factors that influence whether dopamine neuron numbers and tissue dopamine levels are affected by the +/− genotype. Understanding the interaction between how the environment or genetic background can interact with the Nurr1 +/− genotype could have important implications for understanding the genetic complexity of Parkinson’s disease. The interaction between aging and the +/− genotype suggest that aging is an important factor for the regulation of Nurr1 and the function of the mesoaccumbens dopamine system, which could have implications to other neurological problems such as psychosis, addiction or attention deficit hyperactivity disorder in which the mesoaccumbens dopamine neurotransmission has a prominent role.

## Figures and Tables

**Table 1:** Regional dopamine metabolite levels and dopamine turn over.

Striatum	Young +/+	Young +/−	Aged +/+	Aged +/−
**DOPAC**	2.28 ± 0.26	2.61 ± 0.31	1.98 ± 0.29	2.45 ± 0.62
**HVA**	64.30 ± 3.43	50.16 ± 5.26	49.58 ± 4.10	53.11 ± 2.31
**DOPAC/Dopamine**	0.00236 ± 0.00026	0.00296 ± 0.00040	0.00361 ± 0.00110	0.00297 ± 0.00057
**HVA/Dopamine**	0.067 ± 0.004	0.057 ± 0.005	0.077 ± 0.006	0.068 ± 0.007
**Nucleus Accumbens Shell**				
**DOPAC**	2.60 ± 0.83	94 ± 0.50	2.84 ± 0.47	3.67 ± 0.44
**HVA**	32.76 ± 1.83	29.10 ± 1.61	31.37 ± 3.38	28.27 ± 2.92
**DOPAC/Dopamine**	0.00543 ± 0.00127	0.00553 ± 0.00070	0.00579 ± 0.00205	0.01077 ± 0.00306
**HVA/Dopamine**	0.31 ±0.044	0.25 ± 0.029	0.28 ± 0.018	0.36 ± 0.034#
**Nucleus Accumbens Core**				
**DOPAC**	3.67 ± 1.03	7.54 ± 2.39	1.03 ± 0.96	1.07 ± 0.70#
**HVA**	99.11 ± 6.44	108.41 ± 26.53	82.29 ± 13.16	82.65 ± 7.97
**DOPAC/Dopamine**	0.00399 ± 0.00118	0.00474 ± 0.00125	0.00389 ± 0.00130	0.00366 ± 0.00099
**HVA/Dopamine**	0.15 ± 0.02	0.12 ± 0.01	0.14 ± 0.01*	0.23 ± 0.02*,#
**Prefrontal Cortex**				
**Dopamine**	5.51 ± 1.20	4.20 ± 0.850	6.31 ± 1.074	4.85 ± 1.019
**DOPAC**	0.993 ± 0.141	0.898 ± 0.116	0.766 ± 0.107	0.901 ± 0.059
**HVA**	13.07 ± 1.477	11.23 ± 1.774	10.57 ± 1.542	9.00 ± 1.116
**DOPAC/Dopamine**	0.214 ± 0.072	0.282 ± 0.073	0.112 ± 0.015	0.197 ± 0.027
**HVA/Dopamine**	3.175 ± 0.985	2.816 ± 0.452	1.484 ± 0.154	1.818 ± 0.290

* Genotype difference,

# Age difference after ANOVA and post-hoc comparison; p<0.05

**Table 2:** Stereological estimation of regional tyrosine hydroxylase immunoreactive neurons.

	Young +/+	Young +/−	Aged +/+	Aged +/−
**Subtantia nigra**	42.085 ± 3.538	39.717 ± 4.322	37.933 ± 2.814	41.695 ± 4.236
**Ventral tegmental area**	30.982 ± 4.907	35.705 ± 2.703	38.309 ± 1.935	30.774 ± 6.153

Estimates of Tyrosine hydroxylase immunoreactive neurons in the Substanstia nigra and Ventral tegmental area of young and aged +/+ and +/− mice using unbiased stereology. Results are expressed as mean ± SEM
